# Effects of Perceptual Learning Through Binocular Virtual Reality Exercises on Low Stereopsis

**DOI:** 10.3390/jcm15010006

**Published:** 2025-12-19

**Authors:** María Teresa Calderón-González, Irene Sánchez-Pavón, Juan A. Portela-Camino, Santiago Martín-González

**Affiliations:** 1 Servicio de Oftalmología, Hospital Vital Álvarez Buylla, 33611 Mieres, Spain; mariateresa.calderon@sespa.es; 2 Departamento de Óptica, Facultad de Ciencias, Universidad de Granada, 18071 Granada, Spain; 3 Departamento de Física Teórica, Atómica y Óptica, Universidad de Valladolid, 47005 Valladolid, Spain; irene.sanchez.pavon@uva.es; 4 Instituto Universitario de Oftalmobiología Aplicada (IOBA), Universidad de Valladolid, 47005 Valladolid, Spain; 5 Department of Optometry, Begira Ophthalmologic Clinic, 48001 Bilbao, Spain; juanportel@hotmail.com; 6 Department of Construction and Manufacturing Engineering, University of Oviedo, 33204 Gijon, Spain

**Keywords:** amblyopia, strabismus, stereoacuity, perceptual learning, virtual reality

## Abstract

**Objectives**: To evaluate the feasibility and efficacy of a brief, scaffolded virtual reality (VR)-based perceptual-learning protocol to improve stereovision in adults without measurable baseline global stereopsis. **Methods**: Prospective interventional pilot study with a normal-vision reference group. Fourteen adults were enrolled; nine with nil global stereoacuity formed the intervention group and five with normal binocular vision served as controls. Intervention participants completed 8–10 VR sessions (~30 min each over two weeks) using a disparity-driven depth-matching task with individualized thresholds, virtual prism compensation, and interocular contrast balancing. Primary outcomes were changes in clinical stereoacuity (local and global) and Binocular Function (BF). Secondary outcomes included positive fusional vergence (PFV), binocular imbalance (inter-ocular contrast ratio), and in-game depth discrimination error (arcseconds). **Results**: Global stereoacuity improved with four of nine participants who initially showed no measurable global stereopsis achieving measurable thresholds after training (post: 861.11 ± 537.08 arcsec, IQR = 800.00; *p* = 0.06). Local stereoacuity improved near (pre: 616.67 ± 532.68 arcsec, IQR = 1100.00; post: 361.67 ± 537.11, IQR = 200.00; *p* = 0.03) and at a distance (pre: 715.11 ± 573.54 arcsec, IQR = 1114.00; post: 437.11 ± 510.41, IQR = 405.00; *p* = 0.03). BF score improved from 4.33 ± 0.50 (IQR = 1.00) to 3.51 ± 1.14 (IQR = 1.30) (*p* = 0.03). PFV break at distance increased (pre: 17.83 ± 17.53Δ, IQR = 23.50; post: 24.50 ± 17.12Δ, IQR = 28.50; *p* = 0.04), and the binocular imbalance showed a nonsignificant trend towards improvement (*p* = 0.09). In-game depth error decreased from 752.11 ± 384.50 arcsec (IQR = 553.00) to 221.78 ± 79.12 (IQR = 105.00) (*p* < 0.01). Control participants achieved a mean depth error of 43.00 ± 10.30 arcsec after three sessions. **Conclusions**: A short-dose, individualized VR protocol yielded gains in stereoacuity and binocular function in adults with severe stereodeficiency, with transfer from in-game learning to standard clinical measures. While the sample size was limited, the approach proved feasible and was well tolerated, showing encouraging efficiency versus prior high-dose regimens.

## 1. Introduction

Depth perception, the ability to experience the world in three dimensions, arises from various visual cues. While some are monocular (e.g., perspective, occlusion), others require input from both eyes (e.g., convergence, retinal disparity) [1]. This paper focuses on how we learn to appreciate depth by comparing the retinal images in two eyes, each eye having a different line of sight to the same point in space, i.e., stereopsis. Stereopsis, or binocular depth perception, relies on retinal disparity, which arises from the slight differences between the images projected onto each retina. Normal stereopsis requires proper oculomotor alignment and a functional neural mechanism to fuse the two images and compute depth [2].

Stereopsis plays a crucial role in many aspects of visual perception and visually guided behavior. In individuals with normal binocular vision, depth discrimination thresholds are roughly ten times more precise than when viewing with one eye. Disruption of binocular vision leads to marked deficits in visually guided hand movements and in the visuomotor control of locomotion [2].

Stereoacuity is typically assessed using either contour (local stereopsis) or random-dot (global stereopsis) stereograms. Global stereograms depend solely on binocular disparity and contain no monocular cues, whereas local stereograms include recognizable contours that can be partially interpreted monocularly, potentially confounding the evaluation of true stereoscopic ability. Notably, patients lacking measurable global stereopsis may still demonstrate residual local stereopsis [3]. Consequently, random-dot stereograms at near distance have become the gold standard for clinical stereoacuity assessment.

Strabismus is one of the primary causes of stereodeficiency and stereoblindness [4]. In such cases, ocular misalignment disrupts retinal image correspondence, prompting the brain to suppress input from the deviating eye to prevent diplopia and visual confusion. This suppression leads to the development of a scotoma that compromises binocular integration and depth perception [5,6]. Amblyopic anisometropia can also result in stereodeficiency, as image blur and aniseikonia hinder effective binocular fusion [7].

Most randomized controlled trials (RCTs) have evaluated the restoration of stereovision as a secondary outcome of amblyopia treatment [2]. Dichoptic therapies have shown promise, although improvements in stereoacuity remain inconsistent [8]. To date, only one perceptual learning (PL) approach has demonstrated significant stereoacuity gains as a primary outcome in a RCT [9]. This method employed gamified random-dot global stereograms with adaptive disparity thresholds individualized for each participant. However, all participants were required to exhibit some measurable global stereoacuity at baseline (i.e., <800 arcseconds on the Randot Preschool Stereoacuity Test), limiting its applicability to individuals with profound stereodeficiency or stereoblindness.

Small-scale studies have explored alternative approaches that train local rather than global stereoacuity, thereby allowing the inclusion of stereo-blind patients [10,11]. These approaches commonly employ virtual reality (VR) devices, which enable precise control over depth cue composition (excluding accommodation). This allows for a progressive training strategy, beginning with large disparities and leveraging additional depth cues—such as perspective and motion parallax—to scaffold the development of stereopsis. Moreover, dynamic presentation and motion cues, which are stronger in VR than in conventional displays, are used to reinforce disparity-driven depth perception and minimize monocular inference [12,13]. VR systems also provide a wide field of view (typically around 110°), allowing peripheral visual stimulation, a crucial factor for ocular alignment and binocular fusion [14]. Importantly, VR headsets can function as modern digital synoptophores or major amblyoscopes, facilitating binocular vision in strabismic patients by independently manipulating the images presented to each eye. This enables virtual prism correction for horizontal, vertical, or cyclorotational deviations [14]. In addition, VR allows independent adjustment of luminance and contrast, which helps rebalance binocular input in vision disorders and promotes stable binocular perception [10,11].

This study defines a training protocol for individuals without measurable global stereopsis, using perceptual learning at individualized disparity thresholds. The approach leverages VR-based peripheral stimulation and fusion support—such as prism compensation and contrast balancing—and employs contour-based stereograms designed with precise control over size, intra-figure separation, and motion cues. Additionally, the design incorporates a scaffolded cueing strategy that progressively transitions from enriched environments to disparity-only tasks, intended to engage the binocular disparity mechanism. The aim of this study was to conduct a proof-of-concept feasibility pilot using a novel VR-based stereo training protocol incorporating these principles, and to evaluate its feasibility and outcomes in adults with no clinically measurable baseline stereopsis.

## 2. Materials and Methods

### 2.1. Participants

Participants were recruited from the University of Oviedo and the University of Valladolid. All data were collected following informed consent procedures. The study protocol was approved by the Regional Ethics Committee for Clinical Research (Asturias, Spain) and adhered to the principles of the Declaration of Helsinki.

The inclusion criteria in intervention group were subjects with unmeasurable stereoacuity in random dot format, measured with the Random Dot 1S Stereo Acuity Test, i.e., >500 arcsec (Vision Assessment Corporation, Rolling Meadows, IL, USA). The nil stereoacuity cause was the amblyopia, defined as best-corrected visual acuity (BCVA) of ≤0.1 logMAR in the amblyopic eye and an interocular difference of ≥0.2 logMAR, [15] and/or presence of strabismus with a deviation < 20 prism diopters (Δ), defined as heterotropia at near or distance as measured by the Unilateral Cover Test (UCT) under full optical correction and accommodative stimulus [16]. Subjects with congenital malformations, ocular pathology, diplopia under habitual viewing conditions, prematurity (≥8 weeks), developmental delay, and any diagnosed ocular disease were excluded. In addition, due to physical limitations of the virtual reality headset, individuals with interpupillary distance < 55 mm and/or head circumference < 500 mm were also excluded.

Fourteen volunteers (ages 19 to 58 years; mean age 31.9 ± 13.9 years; 7 males, 7 females) were enrolled in the study. Based on global stereoacuity, five participants with measurable stereoacuity were assigned to the control group, while the remaining nine without measurable stereoacuity formed the intervention group. The control group consisted of healthy subjects with stereoacuity equal or better than 63 arcsec (Random Dot 1S Stereo Acuity Test) and BCVA equal to or better than 0.0 logMAR.

Among the intervention group, six participants had amblyopia, three had anisometropia, four had exotropia (three intermittent, one alternating), and three had esotropia (see Table 1, for detailed binocular evaluations see Table 2). Patients were designated S if they had strabismus, A if they had anisometropia, and M if both conditions coexisted. Previous treatments included refractive correction in all participants, occlusion therapy in four cases, and strabismus surgery in one case.

### 2.2. Clinical Protocol

#### 2.2.1. Clinical Evaluation

Participants were evaluated by licensed optometrists (M.T.C.-G. in Oviedo and I.S.-P. in Valladolid, both co-authors). Two clinical assessments were performed: one prior to the intervention to verify eligibility and establish baseline binocular status, and a second after the intervention to assess outcomes.

The initial visual evaluation included best-corrected visual acuity (BCVA) measured with a LogMAR chart displayed on a polarized screen (Optonet Ltd., Warrington, UK); amplitude of accommodation; accommodative response of each eye using the Monocular Estimation Method (MEM); ocular alignment assessed with the Unilateral Cover Test (UCT) using accommodative stimuli; and the presence of anomalous retinal correspondence, determined by comparing the previously obtained objective angle of deviation with the subjective angle measured using a Maddox rod. Based on this comparison, patients were classified as having Normal Retinal Correspondence (NRC) when both angles coincided; Harmonious Anomalous Retinal Correspondence (HARC) when the subjective angle was zero; or Unharmonious Anomalous Retinal Correspondence (UHARC) when the subjective angle differed from the objective angle but was not zero. The examination also included cycloplegic refraction using an autorefractor (TRK-1P; Topcon Medical Systems Inc., Tokyo, Japan) following instillation of 1% cyclopentolate, and anterior and posterior segment evaluation with slit-lamp biomicroscopy and direct ophthalmoscopy.

#### 2.2.2. Binocular Vision Assessment

Binocular function was evaluated through several standardized methods. Sensory status (fusion, suppression, or diplopia) was assessed using a digital version of the Worth Four Dot Test at distance (Optonet Ltd., Warrington, UK). In strabismic patients, the test was performed without prisms to allow detection of spontaneous diplopia.

Positive and negative fusional vergence ranges were measured at distance (3 m) and near (40 cm) using a prism bar, with both break and recovery points recorded in prism diopters. Binocular contrast imbalance was evaluated using a virtual reality test (Dominance; VisionaryTool S.L., Gijón, Spain) [17]. Binocular contrast imbalance quantifies suppression in amblyopia by determining the contrast ratio that “balances” input from the two eyes to the primary visual cortex. For example, a ratio of 2.0 means the non-dominant eye requires twice the contrast of the dominant eye. In the virtual reality test used here, dichoptic letters filtered to a specific spatial frequency are presented, and their contrast is adjusted until the participant reports equal likelihood of perceiving letters in either eye.

Stereoacuity was evaluated using both global and local tests. Global stereoacuity (cyclopean stereopsis) was assessed near using the Random Dot 1S Stereo Acuity Test (Vision Assessment Corporation, Rolling Meadows, IL, USA). This stereogram provides no monocular cues to the depicted objects and includes disparity thresholds of 500, 250, 125, and 63 arcsec. Local stereoacuity (contour stereopsis, which includes monocular cues) was assessed at both near and distance using a computerized contour-based test (Optonet Ltd., Warrington, UK). This test displays four vertical lines, one of which appears in front of the screen plane when viewed with anaglyph glasses. Stimulus dimensions are as follows: line height equivalent to a 1.30 logMAR letter (0.05 decimal acuity), line thickness equivalent to 0.3 logMAR (0.5 decimal), and inter-line spacing equal to four times the line thickness. Depending on screen size, resolution, and viewing distance, stereoacuity thresholds range from 1000 to 10 arcseconds. For patients with no measurable stereopsis, a value of 1300 arcsec was assigned [18].

#### 2.2.3. Intervention

**Figure 1:**
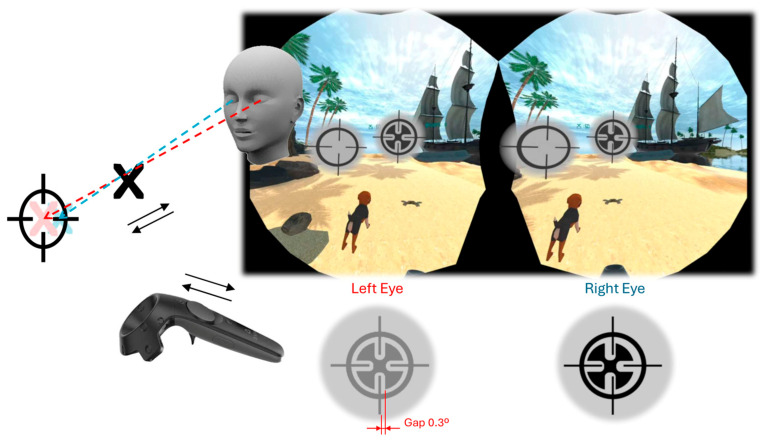
Pirate Island VR game. Participants wear VR headsets and find themselves immersed in an island environment. Their task is to retrieve objects hidden in the sand in the sequence they were lost, serving as a visual memory exercise for gamification purposes. Objects are recovered by aligning in 3D a cross-shaped cursor with a circular target using the controller.

Following informed consent, participants in the intervention group underwent 7 to 10 training sessions over a two-week period using Pirate Island, a virtual reality-based stereopsis training game (Figure 1). Each session lasted approximately 30 min. Control group participants completed only three sessions to establish a reference baseline for performance.

Pirate Island is a gamified, perceptual learning-based VR task developed by VisionaryTool (Spain) in collaboration with the IdeasCAD research group at the University of Oviedo. The game was deployed on the HTC VIVE Pro Eye 2 headset (HTC Corporation, Taoyuan, Taiwan), operating within the SteamVR environment.

The game integrates a visual memory component (to enhance engagement) with a depth discrimination task grounded in binocular disparity cues. Participants are tasked with aligning a movable cross—controlled via hand-tracking or handheld controller—with a fixed circular target in 3D space. The cross can be adjusted in all three spatial dimensions to match the perceived depth of the target. After each trial, the system computes the depth error in arcseconds between the user-controlled cross and the target location. Error values can range from 0 up to >1200 arcsec.

To ensure reliance on binocular disparity, a cue scaffolding strategy is implemented: monocular depth cues are available only during the initial level of each session and are then removed. After a few trials, both the cross and target maintain a constant angular size regardless of simulated depth, effectively eliminating size cues. Cross and target are rendered on a neutral gray background, facilitating adjustment of interocular contrast based on the Dominance test results [17]. Therefore, when a valid result is obtained, the exercise is performed under binocular conditions. This aid is provided only during the initial training sessions, until the participant can successfully complete the 3D task independently. To facilitate stereoscopic depth perception, when the cross is correctly aligned and centered on the target, the resulting gap between them is 0.3° of visual angle, corresponding to peak stereoscopic sensitivity [19,20,21]. Task difficulty is dynamically adjusted based on user performance. Early trials tolerate large depth errors to provide positive reinforcement. As performance improves, the system incrementally reduces the allowed error margin and the stimulus size (carrier spatial frequency), ranging from 30° to as small as 10° of visual angle.

For strabismic participants, a virtual prism was applied to compensate for horizontal, vertical, or cyclotorsional deviations through software-based manipulation, using values obtained with a virtual reality application (Sinoptophore; VisionaryTool S.L., Gijón, Spain). The application functions similarly to a traditional synoptophore or major amblyoscope within a virtual environment, incorporating eye-tracking technology. Virtual prism values correlate with clinically measured deviations, although they are not necessarily identical. In patients with ARC, the subjective angle of deviation is compensated; otherwise, the objective angle is corrected.

All gameplay data are stored securely in the cloud. For each session, the system logs every trial, including target size (in degrees), depth error (arcseconds), and the cross-to-target spatial coordinates for each eye (in degrees of visual field).

### 2.3. Statistical Analysis

Statistical analyses were conducted using SPSS version 26.0 (SPSS Inc., Chicago, IL, USA). Data distribution was assessed using the Kolmogorov–Smirnov test and was found to violate the assumption of normality (*p* < 0.05). Results are presented as mean ± standard deviation (SD) and as medians and interquartile ranges (IQRs). The Wilcoxon signed-rank test was used to compare pre- and post-intervention values.

The effectiveness of the proposed treatment was evaluated through improvements in binocular vision. To more precisely characterize changes in binocular performance, we used the Binocular Function (BF) score to quantify the degree of binocularity, following the approach described by Webber et al. [22]. BF is a unitless binocularity scale that extends stereoacuity measures in cohorts where nil stereoacuity is common, using Worth 4 Dot outcomes. When stereoacuity is present, BF equals stereoacuity in log arc seconds. For nil stereoacuity, a score of 4 is assigned when the Worth 4 Dot indicates simultaneous perception or second-degree fusion, and a score of 5 when it indicates suppression. This scoring method allowed inclusion of all participants in the BF analysis, effectively extending the stereoacuity scale to account for the presence or absence of suppression.

## 3. Results

**Figure 2:**
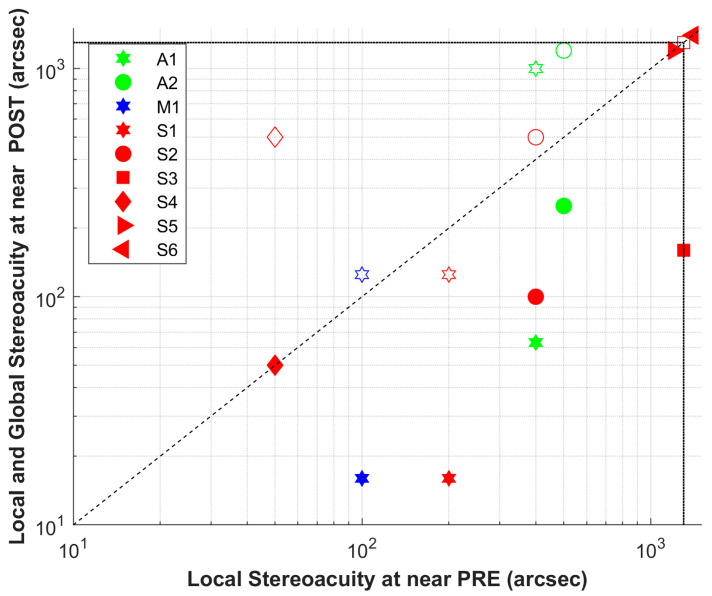
Local stereoacuity at distance pre/post intervention (arcseconds). Points below the 45° reference line represent improvement. Post intervention global stereoacuity is also represented (open symbols). Patients were designated S if they had strabismus (red), A if they had anisometropia (green), and M if both conditions coexisted (blue).

**Figure 3:**
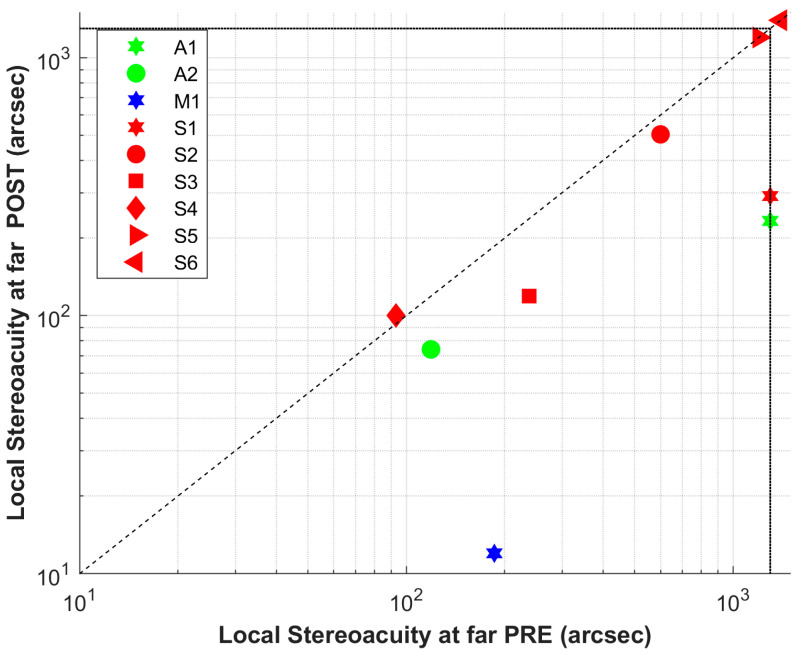
Local stereoacuity at near distance pre/post intervention (arcseconds). Points below the 45° reference line represent improvement. Patients were designated S if they had strabismus (red), A if they had anisometropia (green), and M if both conditions coexisted (blue).

Pre- and post-intervention binocular assessment results are presented in Table 2 and Table 3, respectively. Of the nine volunteers with no measurable global stereoacuity at baseline, four achieved measurable values after the intervention (post: 861.11 ± 537.08, IQR = 800.00; *p* = 0.06). Interestingly, A1 reports perceiving a protrusion in the random-dot pattern but is unable to identify its shape. Regarding near local stereoacuity, one of the three volunteers (S3, S5, and S6) without measurable values at baseline (S3) acquired measurable stereoacuity following the intervention (pre: 616.67 ± 532.68 arcsec, IQR = 1100.00; post: 361.67 ± 537.11, IQR = 200.00; *p* = 0.03). Finally, distance local stereoacuity was acquired by two of the four stereo blind subjects at baseline (A1, S1, S5, and S6)—specifically A1 and S1—showing the same positive trend when considering the entire group (pre: 715.11 ± 573.54 arcsec, IQR = 1114.00; post: 437.11 ± 510.41, IQR = 405.00; *p* = 0.03). Detailed results are shown in Figure 2 (near distance, local and global stereoacuity) and Figure 3 (distance, local stereoacuity). All other descriptive data can be found in Table 4.

Binocular function score improved significantly (pre: 4.33 ± 0.50, IQR = 1.00; post 3.51 ± 1.14, IQR = 1.30; *p* = 0.03). Binocular imbalance improved, but not significantly (pre: 4.76 ± 4.52, IQR = 5.30; post: 3.26 ± 2.76, IQR = 1.62; *p* = 0.09).

Positive fusional vergences at distance also improved significantly (break point, from 17.83 ±17.53 to 24.50 ± 17.12 prismatic diopters, *p* = 0.04).

**Figure 4:**
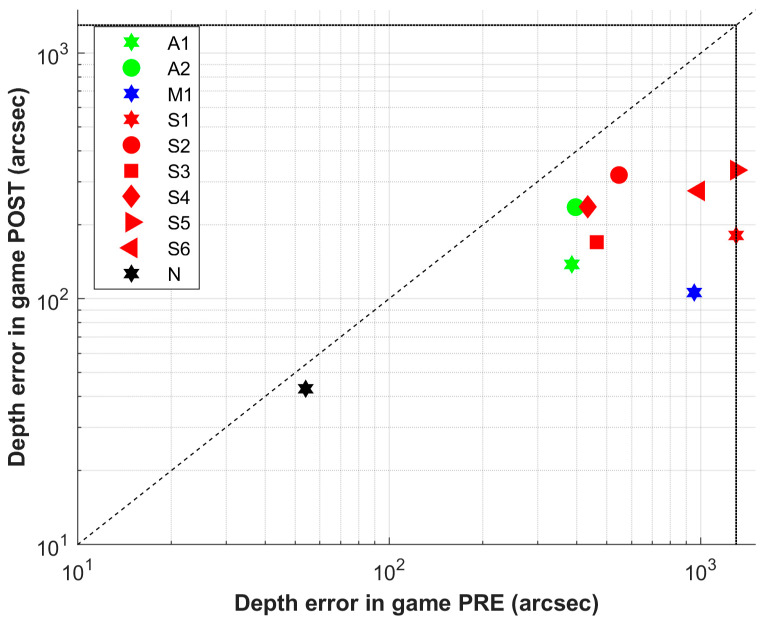
Within Pirate Island game pre/post mean depth judgment error obtained in first and last session of training, in seconds of arc. Points below the 45° reference line represent improvement. Patients were designated S if they had strabismus (red), A if they had anisometropia (green), and M if both conditions coexisted (blue). Control group volunteers achieved a mean depth perception score of 43 arcsec after three sessions, starting from 54 arcsec (N letter, black symbol).

**Figure 5:**
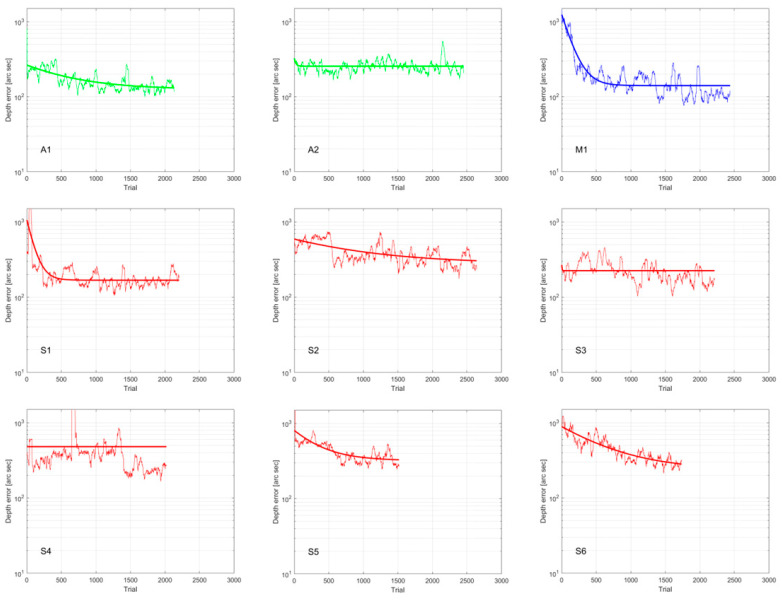
Depth judgment error evolution in logarithmic scale as measured by Pirate Island, on a trial-to-trial basis (data aggregated in groups of 40 trials to smooth graph). The graph shows for each participant the results obtained on each trial, expressed as an error when positioning the cross over the circular sight. An interpolated exponential curve has been added. Depth error could be theoretically zero, although control group measurements showed a lower limit of 43 arcseconds. Graph is limited to 1500 arc seconds error, although within game error can be much bigger. Patients were designated S if they had strabismus (red), A if they had anisometropia (green), and M if both conditions coexisted (blue).

Average depth-judgment errors, as measured by the game, improved from the first to the last training session (pre: 752.11 ± 384.50 arcsec, IQR = 553.00; post: 221.78 ± 79.12, IQR = 105.00; *p* < 0.01) (Table 5 and Figure 4). Depth error evolution during intervention, per volunteer, on a trial-to-trial basis, grouping every 40 trials with the mean value to smooth the graph, are shown in Figure 5. All volunteers completed the treatment, with a mean duration of 8.33 ± 1.73 sessions, 4.03 ± 0.95 h, and 2190.22 ± 355.54 trials.

Control group participants achieved a game depth error of 43.00 ±10.30 arc seconds, considered the reference baseline for within game performance. Details of the optometric and binocular evaluations, as well as the game data, can be found in the Appendix A Table A1, Table A2 and Table A3.

## 4. Discussion

Improvements in depth perception were evident in in-game performance metrics and, more importantly, these gains transferred to clinical assessments of local stereoacuity—and in several cases, even to global stereoacuity measures. These findings are consistent with previous research employing VR video games and contour-based stereograms [10,11]. Moreover, transfer from local to global stereoacuity has been previously documented [3,23]. A notable distinction of the present study is the significantly reduced training intensity compared to earlier studies. Vedamurthy et al. [10] implemented 35 one-hour sessions, and Godinez et al. [11] conducted 40 sessions of similar duration. By contrast, the present protocol consisted of just 8–10 sessions of 30 min each, totaling less than 5 h.

Four of the nine participants acquired measurable global stereoacuity following the intervention, and a fifth participant reported perceiving a protrusion in the random-dot pattern, although she was unable to identify its shape. In random-dot stereograms, the 3D shape must be inferred solely from disparity, whereas in contour stereograms the shape is already available monocularly—thus, coarse depth perception is a prerequisite for recognizing the embedded shape. The improvement is clinically evident in M1 and S1, who reached 125 arcsec, whereas S2 and S4 both reached 500 arcsec, the largest value on the Random Dot 1S Stereo Acuity Test, making their improvement less certain. Results are consistent with findings by Vedamurthy et al. [10] and Godinez et al. [11] where individuals with no measurable baseline stereoacuity on clinical tests (defined as >400 arcsec on the Randot Circles Stereotest) achieved post-intervention thresholds of 140 arcsec or better.

Game results should be interpreted with caution. Gameplay data are inherently noisy because game mechanics must balance motivation, attention, and task performance. Individual play styles also introduce variability. According to first/last game depth error results (Table 5 and Figure 4), although final depth error values in the intervention group did not reach the precision observed in the control group, depth discrimination performance improved in all participants. Figure 5 provides a detailed view of depth error evolution across sessions. While some participants show a clear trend toward improvement, this is not the case for all. Several participants exhibit considerable fluctuations in their trial-by-trial performance, which may reflect the natural consolidation process of learning or occasional lapses in attention. For A2 and S4 and possibly S3, considering only the first and last sessions could overestimate their improvement. In contrast, the evolution of A1, M1, S1, S2, S5, and S6 is consistent, confirming their significant gains. Moreover, the exponential fit does not show a clear plateau (long enough to allow learning consolidation) for A1, S2, S5, and S6, suggesting that additional sessions could have yielded further improvements.

There is evidence that stereoacuity loss in strabismic patients is more resistant to treatment compared to losses associated with anisometropia [4,24]. In the present study, seven participants had strabismus (three with intermittent exotropia, one with alternating exotropia, and three with esotropia). All but two demonstrated improvements in local stereoacuity. Notably, four developed measurable global stereoacuity, including all three participants with intermittent exotropia (M1, S1, and S2). This may be attributed to intermittent strabismus preserving elements of the neural substrate for binocular fusion, as stereoscopic input is intermittently present despite being abnormal. The same reasoning applies to S5 and S6, who had constant deviations and showed no measurable global stereoacuity after the treatment.

ARC was present in S4 (unharmonious) and S6 (harmonious). Although S4 achieved a final global stereoacuity of 500 arcsec, this outcome cannot be confidently attributed to the treatment, as the erratic depth error pattern (Figure 5) reveals marked binocular instability that undermines the reliability of this apparent improvement. In contrast, while S6 did not achieve global or local stereoacuity, in-game results showed a clear trend toward improvement.

Among participants with strabismus, visual angle deviation decreased at both distance and near fixation in all but S4, S5, and S6. Fusional vergence ranges (both positive and negative) also improved at near and distance fixation. These changes were not reported in the study by Godinez et al. [11] possibly due to their smaller sample size or the lower vergence demand of their training protocol compared to the present study.

Improvements in binocularity were also reflected in performance on the Worth Four Dot Test. When analyzed using the composite Binocular Function (BF) metric, these improvements became even more evident, particularly in participants who developed measurable global stereoacuity.

Baseline binocular imbalance emerged as a potential predictor of training outcome. Participants S3, S5, S6, and A2 exhibited the highest interocular contrast ratios at baseline (ranging from 5 to 12) and were the only individuals who failed to acquire global stereoacuity post-intervention. Furthermore, S5 and S6, who had the highest contrast ratios among them, did not achieve measurable local stereoacuity either. Although all participants demonstrated reductions in contrast ratio following the intervention, S5 and S6 still exhibited the highest post-treatment values. Martin et al. [17] previously suggested that binocular imbalance could serve as a biomarker for tracking recovery of stereovision, even when stereoacuity itself does not measurably improve. To our knowledge, this study is the first to document changes in binocular imbalance during a stereoacuity training protocol.

The VR system used in this study incorporated the baseline imbalance score and adjusted interocular contrast to facilitate binocular viewing. While optimal stereo performance typically occurs when both eyes are presented with identical physical contrast [24], some participants—particularly A2, S3, S5, and S6—required contrast balancing to initiate training. Notably, after a few sessions, all participants preferred to train without the contrast filter, supporting the idea that stereoacuity benefits from binocular equality once fusion is achievable.

Participants A2 (37 years), S5 (53 years), and S6 (58 years) were the oldest individuals in the sample. Stereoacuity treatment in adulthood is seldom attempted, as diminished neuroplasticity is believed to limit efficacy. However, growing evidence suggests that stereovision can be improved well beyond the critical period [4,25,26,27]. All participants in this study were outside the typical sensitive period. A2, S5, and S6 also presented with high binocular imbalance; S5 and S6 had a history of occlusion therapy, and S6 additionally demonstrated anomalous retinal correspondence, collectively suggesting poor prognosis for rehabilitation.

Nonetheless, both game performance and reductions in binocular imbalance indicate that at least participants S5 and S6 may have benefited from a longer intervention. As previously noted, the total intervention duration in this study was less than five hours, substantially less than in previous protocols.

The treatment was performed in adult subjects with no measurable stereopsis at baseline, and four out of nine achieved measurable stereoacuity on clinical testing. This improvement could subsequently allow these patients to undergo direct stereopsis stimulation therapies, as described in several previous studies using visual training or stereoscopic video game-based approaches [9,25,26,27].

### Study Limitations

This study has several limitations that should be considered. First, the small sample size and the heterogeneity of the underlying binocular disorders limit the generalizability of the findings; therefore, the results should be interpreted as exploratory. Larger controlled studies will be required to determine the efficacy of the protocol within specific diagnostic subgroups. Second, the absence of a placebo or sham control group restricts the strength of the conclusions. Without a control condition, it is difficult to determine whether the observed improvements are attributable solely to the intervention or whether they may be influenced by nonspecific factors such as practice or learning effects. Third, neither the examiners nor the participants were masked to the intervention. Because stereoacuity assessments and fusional vergence measurements include subjective components, awareness of receiving active treatment may have introduced placebo or expectation bias, particularly in the local stereoacuity and PFV outcomes. Although the primary aim of this work was feasibility rather than definitive efficacy, these factors may have contributed to some of the observed improvements. Future randomized, masked, sham-controlled trials with larger samples will be essential to minimize these biases and isolate the specific therapeutic effect of the proposed VR-based training.

## 5. Conclusions

A brief, scaffolded, VR-based perceptual-learning protocol incorporating individualized disparity thresholds, virtual prism compensation, and dynamic contrast balancing improved depth discrimination and clinical stereoacuity in adults without measurable baseline global stereopsis. Gains in local stereoacuity were common, and several participants achieved measurable global stereopsis. These findings support VR-enabled, cue-scaffolded training as a feasible and potentially efficient approach for rehabilitating stereovision in adults with severe stereodeficiency.

## Figures and Tables

**Table 1:** Intervention group optometric evaluations.

ID	Sex	Age	Rx	BCVA	AA	MEM	Prev. Tx	Diagnosis
A1	F	19	+0.50/+1.50 −0.50@180°	1.25/0.9–2	10/6.66	+0.25/+0.75	Occlusion	Amb. Aniso
A2	M	37	+4.50 −3.00@20°/0.00	0.8/1.2	6.75/8.25	+3.00/+0.00		Amb. Aniso
M1	F	19	+1.50 −0.50@170°/+4.75 −1.25@175°	1.6/0.8 + 3	12.5/8.33	+1.25/+2.00	Occlusion	Amb. Aniso XT Int.
S1	F	20	−1.00/+0.25 (Overminus −1.50)	1.0/1.0	10/10	−0.25/+0.25		XT int.
S2	M	27	+3.75 +2.00@90°/+2.50 +1.75@70°	1.0/1.0	15/15	+0.75/+1.25		XT int
S3	F	19	+7.00 −2.50@20°/+6.50 −3.50@165°	1.0/1.0	15/15	+0.75/+1.25		XT alt.
S4	F	19	+2.50 −1.50@180°/+2.00	0.8/1.26	7.7/7.7	+0.75/+0.75	Sx RE ET	Amb. ET
S5	F	53	+1.25/+0.50	0.8/1.26	presbyopic	+1.00/+1.00	Occlusion	Amb. ET
S6	M	58	+1.00/+2.00	1.25/0.8	presbyopic	+0.75/+1.25	Occlusion	Amb. ET

Sex (M, male; F, female); Age (years); Rx, refraction in diopters; BCVA, best corrected visual acuity (decimal); AA, accommodation amplitude (diopters); MEM, Monocular Estimation Method (diopters, RE and LE); previous treatments and diagnosis (Sx, surgery; RE/LE, right or left eye; ET/XT, eso and exo tropia; Int., intermittent; Alt., alternant; Amb., amblyopia; Aniso, anisometropic).

**Table 2:** Intervention group binocular evaluations pre-treatment.

ID	UCT Distance	UCT Near	RC	FV Distance	FV Near	Worth Test	Ratio	SA Local Distance	SA Local Near	SA Global
A1	3EF	3EF	NRC	NFV 12/6 PFV 18/12	NFV 14/12 PFV 30/20	Sup. LE	1.02	no	400	no
A2	ortho	ortho	NRC	NFV 4/2 PFV 10/8	NFV 14/12 PFV -/-	Fusion	6.43	119	500	no
M1	18XT int.	20XT int.	NRC	NFV 18/16 PFV 6/-	NFV 20/18 PFV -/-	Int. Sup.	2.03	186	100	no
S1	18XT int.	14XT int.	NRC	NFV 20/10 PFV -/-	NFV 25/- PFV > 45	Int. Sup.	1.13	no	200	no
S2	8XT int.	12XT int.	NRC	NFV 10/8 PFV 16/8	NFV 3/0 PFV 18/14	Int. Sup.	1.10	600	400	no
S3	12XT	15XT	NRC	NFV 12/8 PFV 25/16	NFV 30/25 PFV 14/12	Alt. Int. Sup.	5.19	238	no	no
S4	16ET-3HPT ^2^	18ET-2HPT ^2^	UHARC ^2^	no	no	Diplopia	1.95	93	50	no
S5	14 ET	16 ET	NRC	no	no	Sup. RE	12.00	no	no	no
S6	10ET	10ET	HARC	no	no	Sup. LE	12.00	no	no	no
	UCT, Unilateral Cover Test; EF, esophoria; XF, exophoria; ET, esotropia; XT, exotropia; HPF, hyperphoria; RC, retinal correspondence; NRC, normal RC; ARC, anomalous RC; HARC, Harmonious ARC; UHARC, Unharmonious ARC; FV, fusional vergences; NFV/PFV, negative/positive FV; Int., intermittent; Alt., alternant; Sup., suppression; LE/RE, left/right eye; Ratio, binocular contrast ratio; SA, stereoacuity. CT and FV in diopters; SA in seconds of arc; Ratio unitless.									
	^2^ S4 was diagnosed with strabismus at age 3 and underwent surgery at 14. The subjective deviation was 6Δ at both distance and near.									

**Table 3:** Intervention group binocular evaluations post-treatment.

ID	UCT Distance	UCT Near	RC	FV Distance	FV Near	Worth Test	Ratio	SA Local Distance	SA Local Near	SA Global
A1	3EF	3EF	NRC	NFV 25/12 PFV 30/20	NFV 12/10 PFV > 45	Fusion	1.02	233	63	no ^2^
A2	Orto	orto	NRC	NFV 4/2 PFV 8/4	NFV 10/8 PFV 6/4	Fusion	1.68	74	250	no
M1	16XT	18XT inter.	NRC	NFV 16/14 PFV 6/-	NFV 25/20 PFV 10/6	Fusion	1.78	12	16	125
S1	10XF/10XF (OM)	10XF/6XF (OM)	NRC	NFV 16/12 PFV 10/-	NFV 20/16 PFV > 45	Fusion	1.13	291	16	125
S2	6XT int.	3XT int.	NRC	NFV 10/6 PFV 25/18	NFV 8/6 PFV 25/18	Int. Sup.	3.30	505	100	500
S3	6XT	3XT	NRC	NFV 16/18 PFV 10/8	NFV 30/25 PFV 16/12	Alt. Int. Sup.	2.38	119	160	no
S4	16ET-3HPT	18ET-2HPT	UHARC	no	no	Diplopia	2.10	100	50	500
S5	14 ET	16 ET	NRC	no	no	Fusion	7.97	no	no	no
S6	12ET	12ET	HARC	no	no	Sup. LE	7.97	no	no	no
	UCT, Unilateral Cover Test; EF, esophoria; XF, exophoria; ET, esotropia; XT, exotropia; HPF, hyperphoria; OM, overminus; RC, retinal correspondence; NRC, normal RC; ARC, anomalous RC; HARC, Harmonious ARC; UHARC, Unharmonious ARC; FV, fusional vergences; NFV/PFV, negative/positive FV; Int., intermittent; Alt., alternant; Sup., suppression; LE/RE, left/right eye; Ratio, binocular contrast ratio; SA, stereoacuity. CT and FV in diopters; SA in seconds of arc; Ratio unitless.									
	^2^ A1 reports perceiving a protrusion in the random-dot pattern but is unable to identify its shape									

**Table 4:** Descriptive data of the intervention group.

	Pre		Post		
Test	Mean ± SD	Median (IQR)	Mean ± SD	Median (IQR)	*p*
BF	4.33 ± 0.50	4.00 (1.00)	3.51 ± 1.14	4.00 (1.30)	0.03 *
Ratio	4.76 ± 4.52	2.03 (5.30)	3.26 ± 2.76	2.10 (1.62)	0.09
Depth Error	752.11 ± 384.50	547.00 (553.00)	221.78 ± 79.12	236.00 (105.00)	<0.01 *
		Near			
SA global	1300 ± 0.00	1300.00 (0.00)	861.11 ± 537.08	1300.00 (800.00)	0.06
SA local	616.67 ± 532.68	400.00 (1100.00)	361.67 ± 537.11	100.00 (200.00)	0.03 *
UCT	15.00 ± 3.42	15.00 (4.00)	11.43 ± 6.48	12.00 (10.50)	0.10
NFV Break	12.67 ± 5.75	12.00 (6.00)	14.50 ± 7.04	16.00 (4.50)	0.58
NFV Recovery	8.33 ± 4.63	8.00 (3.00)	10.67 ± 5.75	12.00 (6.00)	0.34
PFV Break	12.50 ± 8.98	13.00 (10.50)	14.85 ± 10.05	10.00 (12.75)	0.69
PFV Recovery	7.33 ± 6.41	8.00 (9.00)	8.33 ± 8.80	6.00 (14.50)	0.58
		Distance			
SA local	715.11 ± 573.54	600.00 (1114.00)	437.11 ± 510.41	233.00 (405.00)	0.03 *
UCT	13.71 ± 3.90	14.00 (6.00)	11.43 ± 4.28	12.00 (7.00)	0.13
NFV Break	17.67 ± 9.52	17.00 (9.75)	17.50 ± 8.89	16.00 (13.25)	0.89
NFV Recovery	11.71 ± 9.89	12.00 (13.50)	14.17 ± 7.44	13.00 (10.50)	0.42
PFV Break	17.83 ± 17.53	16.00 (23.50)	24.50 ± 17.12	20.50 (28.50)	0.04 *
PFV Recovery	15.17 ± 16.64	13.00 (15.50)	21.67 ± 18.67	15.00 (30.75)	0.06

SD, Standard deviation; IQR, interquartile range; BF, Binocular function; Ratio, Binocular contrast ratio; Depth error, mean disparity-judgment error calculated by the game (first and last sessions); SA, Stereoacuity; UCT, Unilateral Cover test; NFV, Negative fusional vergence; PFV, Positive fusional vergence; *p*, *p*-value market with * if significant. UCT and FV in diopters; SA and Depth error in seconds of arc; Ratio unitless.

**Table 5:** Intervention group Pirate Island game data.

**ID**	**Hours**	**Sessions**	**Days**	**Total Trials**	**Initial Error ^1^**	**Final Error ^1^**
A1	3.06	7	12	2173	386	138
A2	4.01	8	14	2492	397	236
M1	3.51	8	10	2478	954	106
S1	3.72	9	29	2244	1300	181
S2	4.20	6	21	2680	547	319
S3	4.10	7	31	2252	464	170
S4	2.98	8	22	2061	434	237
S5	4.56	11	23	1556	1300	334
S6	6.15	11	24	1776	987	275

^1^ Within game pre/post mean depth judgment error obtained in first and last session of training, in seconds of arc.

## Data Availability

The original contributions presented in this study are included in the article. Further inquiries can be directed to the corresponding author.

## Abbreviations

The following abbreviations are used in this manuscript:

VR	Virtual Reality
RTC	Randomized Controlled Trial
BCVA	Best-Corrected Visual Acuity
BF	Binocular Function
UCT	Unilateral Cover Test
FV	Fusional Vergence
PFV/NFV	Positive/Negative FV
RC	Retinal Correspondence
NRC	Normal RC
ARC	Anomalous RC
HARC	Harmonious ARC
UHARC	Unharmonious ARC
SA	Stereoacuity
LE/RE	Left/Right Eye

## Appendix A

**Table A1:** Control group optometric evaluations.

ID	Sex	Age	Rx	AA	MEM	Notes
N1	M	35	−1.50/−1.75	5/5	+1.0/+1.0	Overcorrected with negative lens
N2	F	52	−1.50/−1.75/Ad +2.00	-	-	Presbyopic
N3	M	23	−2.25 −0.50 95°/−1.50 −1.25 90°	20/20	+0.50/+0.50	Protanomalous
N4	M	44	+0.25/+0.50	6.66/5	+0.50/+1.0	
N5	M	22	+1.25/+1.25	6.66/6.66	+1.00/+1.00	

Sex (M, male; F, female); age (years); Rx, refraction in diopters; AA, accommodation amplitude (diopters); MEM, Monocular Estimation Method (diopters).

**Table A2:** Control group binocular evaluations.

ID	UCT Distance	UCT Near	FV Distance	FV Near	Worth Test	Contrast Ratio	SA Local Distance	SA Global
N1	2EF	ortho	PFV 30/25 NFV 6/2	PFV 45/40 NFV 14/10	Fusion	1.05	23	63
N2	2EF	12EF	PFV 25/20 NFV 4/1	PFV 45/35 NFV 12/10	Fusion	1.24	163	63
N3	ortho	2XF	PFV 25/20 NFV 8/4	PFV 25/20 NFV 16/12	Fusion	1.32	47	63
N4	ortho	ortho	PFV 25/16 NFV 10/6	PFV > 45 NFV 20/18	Fusion	1.22	163	63
N5	2XF	6XF	PFV 16/12 NFV 10/6	PFV 30/25 NFV 20/14	Fusion	1.32	12	63

UCT, unilateral cover test; EF, esophoria; XF, exophoria; FV, fusional vergences; NFV/PFV, negative/positive FV; SA, stereoacuity in arcseconds.

**Table A3:** Control group Pirate Island game data.

ID	Hours	Sessions	Days	Initial Error	Final Error
N1	1.32	3	3	25	36
N2	1.31	3	3	98	56
N3	1.30	3	3	31	38
N4	1.31	3	3	36	33
N5	1.30	3	3	80	52

Within game pre/post depth perception error measured in arcseconds.
